# Increasing inflationary T-cell responses following transient depletion of MCMV-specific memory T cells

**DOI:** 10.1002/eji.201445016

**Published:** 2014-10-20

**Authors:** Stuart Sims, Paul Klenerman

**Affiliations:** Peter Medawar Building for Pathogen Research, University of OxfordOxford, UK

**Keywords:** Inflationary T-cell response, MCMV, Memory T cells

## Abstract

Murine CMV (MCMV) infection induces effector CD8^+^ T cells that continue to increase in frequency after acute infection (“inflation”) and are stably maintained at a high frequency, with up to 20% of the CD8^+^ T-cell compartment being specific for one epitope, although the flexibility and turnover of these populations is not fully defined. Here we report that effector/memory CD8^+^ T cells induced by MCMV can be paradoxically boosted following transient depletion of epitope specific CD8^+^ T cells. Treatment of MCMV-infected mice with MHC-Class I-saporin tetramers led to partial (80–90%) depletion of epitope-specific CD8^+^ T cells—rapidly followed by a rebound, leading to expansion and maintenance of up to 40% of total CD8^+^ T cells, with minimal changes in response to a control epitope (M45). These data indicate the tight balance between host and virus during persistent infection and the functional flexibility of the “inflated” CD8^+^ T cell responses during persistent infection.

## Introduction

Conventional CD8^+^ T-cell response to infections result in a rapid proliferation then contraction upon clearance. However, murine cytomegalovirus (MCMV) infection leads to life-long latency, with sporadic low-level replication [1]. As the virus establishes latency, limited immunodominant epitopes arise. These MCMV-specific cells accumulate over time, display “effector memory” (T_EM_) phenotypes [2] and eventually stabilize at a high frequency [3]. This “memory inflation” is in contrast to the immunodominant epitope-specific CD8^+^ T-cell response from the acute phase (M45) that shows a classical memory response and a “central memory” phenotype.

The inflationary CD8^+^ T-cell phenotype suggests that their accumulation and maintenance is driven by viral antigen [1]. However, in contrast to chronic LCMV infection, where CD8^+^ T cells become exhausted due to repetitive stimulation (e.g. upregulation of the exhaustion marker PD1 and loss of IFN-γ secretion/proliferative function) [4], this is clearly not the case with inflationary CD8^+^ T cells that are still functional during chronic infection [5].

It has previously been suggested that inflationary CD8^+^ T cells are maintained by the recruitment of precursors ± naïve CD8^+^ T cells [6,7]. However, many questions still remain as to their stability and kinetics of their turnover. To analyze this we used a method to specifically delete antigen-specific CD8^+^ T cells in vivo. MHC class I tetramers providing specific toxin delivery are produced by coupling biotinylated MHC-peptide monomers with streptavidin bound to saporin (Supporting Information Fig. 1) [8,9]. This approach has been used to delay or prevent T-cell mediated disease [10,11]. Here we address the effect of transient depletion of virus-specific CD8^+^ T-cell populations. Surprisingly, we found that the inflationary (but not noninflationary) CD8^+^ T cells rebound six days postdepletion, reaching an elevated frequency at which they are sustained as a new set-point. This reveals the tight host–virus balance in persistent infections, and the in vivo functionality of “inflationary” T-cell responses [12].

## Results and discussion

**Figure 1:**
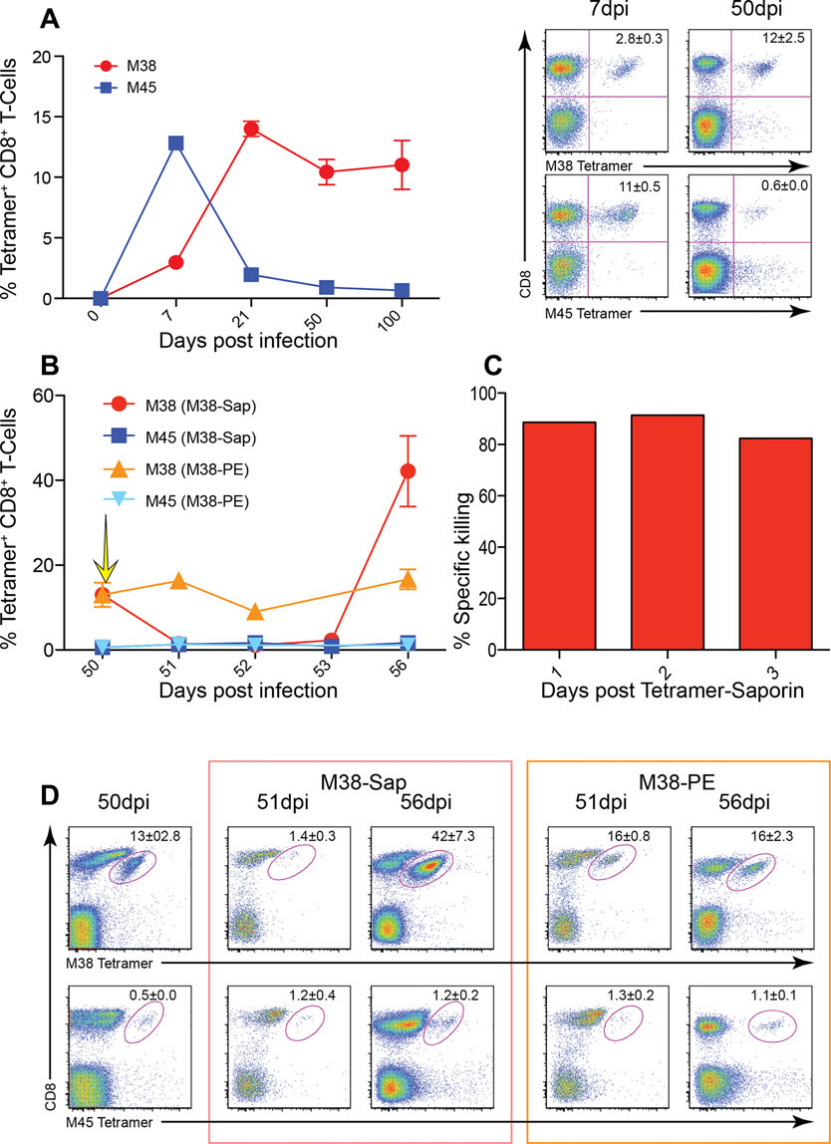
Frequency and function of MCMV-specific CD8^+^ T cells and depletion of M38-specific CD8^+^ T cells. C57BL/6 mice were infected intravenously (i.v.) with 1 × 10^6^ pfu MCMV. (A) Time course for M38- (red) and M45- (blue) specific CD8^+^ T cells. Lymphocytes from 0, 7, 21, 50, and 100 days postinfection mice were stained with tetramers, and analyzed by flow cytometry. Right panel: representative flow cytometry plots showing tetramer^+^ CD8^+^ T lymphocytes are shown (*n* = 8/group). (B) Fifty days postinfection, mice were injected with either M38-teramer-saporin or M38-tetramer-PE. Yellow arrow indicates time point of M38-Saporin injection. Time course for M38- (red) and M45- (blue) specific CD8^+^ T cells after injection with M38-teramer-saporin, and M38- (orange) and M45- (light blue) specific CD8^+^ T cells after injection with M38-tetramer-PE. Mice were bled 0, 1, 2, 3, and 6 days postinjection, stained with tetramers, and analyzed by flow cytometry. (A and B) Data represent mean percentages of live tetramer^+^ CD8^+^ T lymphocytes (mean ± SEM) of (A) *n* = 8 or (B) *n* = 6/group and are pooled from two independent experiments. (C) The percentage killing of M38-specific CD8^+^ T cells after injection with M38-teramer-saporin analyzed by flow cytometry. Data represent mean percentages of specific killing of tetramer^+^ CD8^+^ T lymphocytes (*n* = 6/group) and are pooled from two independent experiments. (D) Representative flow cytometry plots of M38-specific CD8^+^ T cells and M45-specific CD8^+^ T cells 50 days after MCMV infection, 1 and 6 days after either M38-tetramer-saporin (red box) or M38-tetramer-PE injection (orange box). Data are shown as mean ± SEM and are from one representative plot out of 6/group).

Infection of C57BL/6 with MCMV induces two types of CD8^+^ T-cell responses; staining with MHC-peptide tetramers shows that M45-specific CD8^+^ T cells are immunodominant in acute infection, reaching frequencies of 11% of CD8^+^ T cells, and then sharply declining to 0.6% CD8^+^ T cells. In comparison, the inflationary M38-specific response gradually increases, reaching 12% of CD8^+^ T cells in blood, and is maintained thereafter at this high frequency (Fig.1A).

To investigate the stability of the inflationary CD8^+^ T-cell population, mice 50 days (d) postinfection were treated with 44ρM of saporin-conjugated M38-tetramer to target the specific CD8^+^ T-cell population (Fig.1B). One day post-treatment specific killing of 88% of M38-specific CD8^+^ T cells was achieved (Fig.1C), i.e. a decrease from 13 to 1.4% M38-specific CD8^+^ T cells in blood, and maintained for a further 2 days. Surprisingly, after 6 days the percentage of M38-specific CD8^+^ T cells had returned, and thereafter increased, with 42% of CD8^+^ T cells M38-specific. In parallel, we saw no decrease in M45-specific CD8^+^ T cells following M38-tetramer treatment (0.6% of CD8 T cells predepletion, 1.2% 1 day postdepletion) and no subsequent expansion was observed (1.2% 6 day postdepletion; Fig.1D). As a further control, injection with phycoerythrin (PE)-conjugated M38-tetramer showed a small modulation of M38-specific CD8^+^ T cells rising from 13 to 16% (Fig.1D) without further change.

We tested whether the effects seen were due to redistribution of epitope-specific cells by analysis of organs over time. The partial depletion and subsequent increase in the percentage of M38-specific CD8^+^ T cells was not restricted to the blood, but was also observed in the spleen, liver and lung (Supporting Information Fig. 2). This was most marked in the lung and liver, with, respectively, an increase to 51 and 42% of M38-specific CD8^+^ T cells 6 days post-treatment. There was no change in the percentage of M38-specific CD8^+^ T cells after the injection with M38-tetramer-PE in any of the organs, and minimal change seen in M45 populations in the organs (Supporting Information Fig. 2).

**Figure 2:**
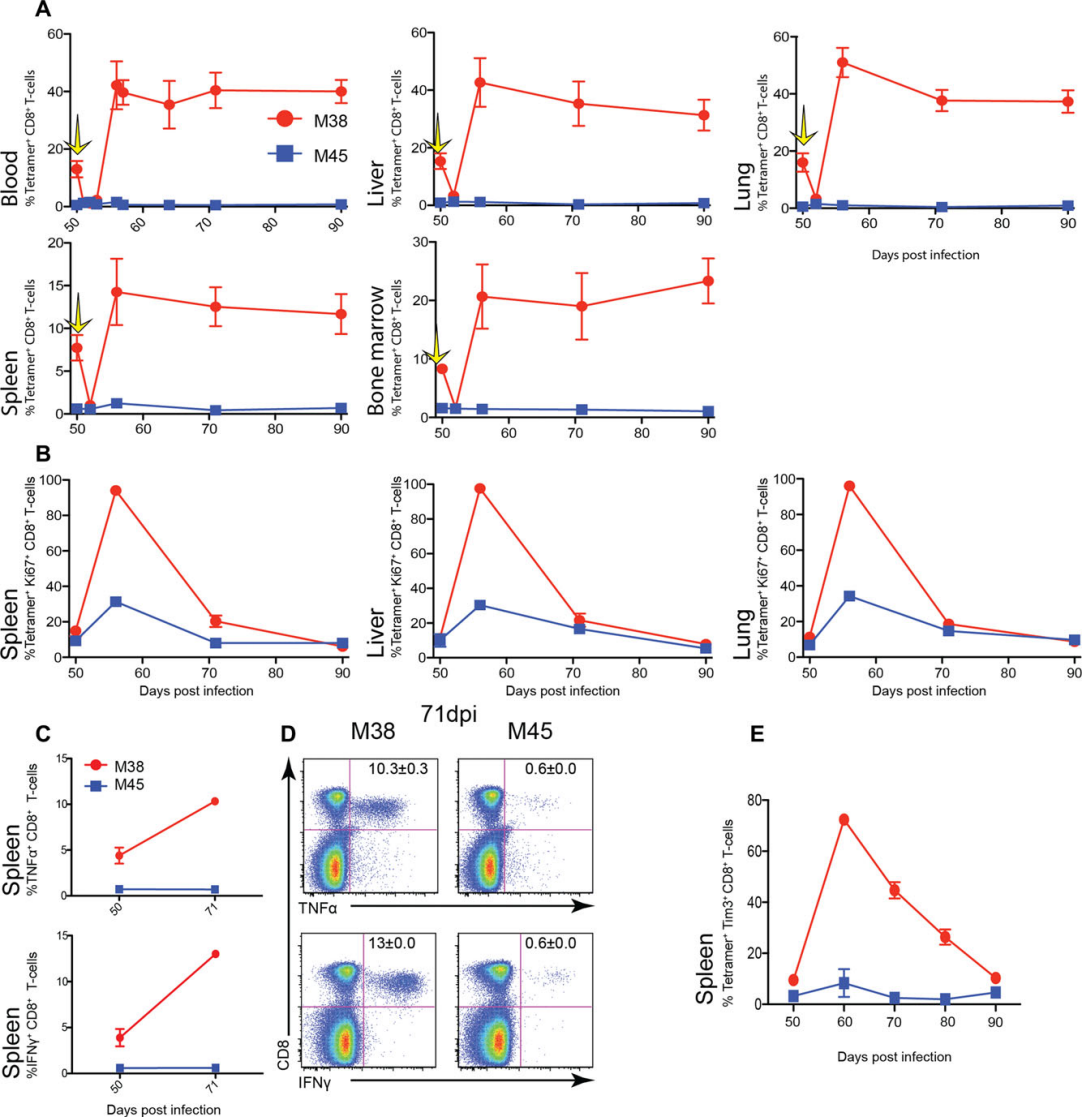
Increased frequency of M38-specific CD8^+^ T cells after depletion. C57BL/6 mice were infected (i.v) with 1 × 10^6^ pfu MCMV. Fifty days postinfection, mice were injected with M38-teramer-saporin, lymphocytes harvested from the blood, liver, lung, spleen, and bone marrow at 0, 2, 6, 21, and 40 days postinjection stained with tetramers, and analyzed by flow cytometry. (A) Time course showing mean percentages of live tetramer^+^ CD8^+^ T lymphocytes isolated from the blood, liver, lung, spleen, and bone marrow. Yellow arrow indicates time point of M38-Saporin injection. (B) Time course displaying the percentage of cells expressing Ki67 on M38- CD8^+^ T cells (red) and M45-specific CD8^+^ T cells (blue) in the spleen, liver and lung at indicated time points postinjection with M38-tetramer-saporin. (C) Change in the percentage of cells expressing IFN-γ, and TNF-α before and 21 days after M38-tetramer-saporin injection. Splenocytes were harvested and stimulated with either M38 or M45 peptide. (D) Representative flow cytometry plots of splenocytes producing IFN-γ and TNF-α stimulated with either M38 or M45 peptide in mice 21 days after M38-tetramer-saporin injection. Numbers indicate the percentage of IFN-γ^+^ CD8^+^ T cells (mean ± SEM). Plots are representative of two independent experiments. (E) Time course showing the percentage of cells expressing Tim3 on M38-specific CD8^+^ T cells (red) and M45-specific CD8^+^ T cells (blue) in the spleen, liver, and lung at indicated time points postinjection with M38-tetramer-saporin. (A–E) Data are shown as mean ± SEM (*n* = 6) and pooled from two independent experiments.

To determine whether this increased frequency of M38-specific CD8^+^ T cells after treatment was stable, mice were treated 50 days postinfection with 44 ρM M38-tetramer-saporin, and frequencies of tetramer-positive cells tracked in organs (Fig.2A). Reduction of M38-specific CD8^+^ T cells was achieved in all organs measured (blood, spleen, liver, lung, and bone marrow) at 2 days; this was then followed by a doubling of the original percentage of M38-specific CD8^+^ T cells across all organs, which was maintained up to 90 days.

The total number of M38-specific CD8^±^ T cells present in the spleen was measured. Two days after injection (p.i.) with M38-tetramer-saporin, a dramatic decrease in the total number of M38-specific CD8^±^ T cells was observed. Four days p.i., M38-specific CD8^±^ T-cell numbers rebounded to levels similar to those seen before injection, and 40 days p.i., the numbers increased even more. (Supporting Information Fig. 3).

To evaluate the proliferative state of the M38-specific T cells postdepletion, staining for Ki67 (Fig.2B) was performed. In the liver, lung, and spleen almost 100% of M38-specific CD8^+^ T cells were positive for the proliferation marker at 6 days, decreasing to a level similar to that before injection by 40 days. A transient increase in M45-specific CD8^+^ T-cell frequencies from 0.6 to 1.5% of CD8^+^ T cells was noted, decreasing to levels observed predepletion. This was accompanied by a transient increase in the proliferation marker Ki67 (from 9 to 30% 6 days.). This transient impact on M45-specific CD8^+^ T cells could be due to a release of immunological pressure on the virus allowing viral transcription to proceed until the next immunological checkpoint is reached [13], or could be due to “off-target” or nonspecific impacts of the therapy.

To discover if these cells displayed any evidence of exhaustion, ICS was performed (Fig.2C). The percentage of cells producing IFN-γ and TNF-α when stimulated with M38 peptide was 4 and 5%, respectively, predepletion, and this rose to 13 and 10% at 21 days. There was no equivalent increase in M45-specific responses.

The phenotype of M38-specific CD8**^+^** T cells postdepletion did not significantly change in the blood or organs (Supporting Information Fig. 4). They remained low in CD62L, CXCR3, CD27, CD127, and CD122 expression in comparison to M45-specific CD8**^+^** T cells. There was a subtle reduction in CD127 expression postdepletion, which reverted after 21 days. High expression of the NK cell receptors NKG2A and NKG2D was maintained. The percentage of M38-specific CD8**^+^** T cells expressing TIM3 increased—nearly 100% of M38-specific cells expressed the marker at 6 days; this then gradually decreased over 40 days to predepletion levels (Fig.2E). This marker correlates with the proliferation state of CD8^+^ T cells and is not uniquely expressed on exhausted cells. Overall the M38-specific CD8**^+^** T cells proliferated actively, maintained their T_EM_ phenotype and did not display any increased signs of exhaustion.

To test the impact of saporin treatment on a noninflating epitope, M45-tetramer-saporin was generated and mice were treated 50 days postinfection. A decrease in M45-specific CD8**^+^** T cells from 0.6 to 0.1% of CD8 T cells was achieved at 2 days. By 6 days the percentage of M45-specific CD8**^+^** T cells had returned to pre-depletion levels and was maintained up to 21 days (Supporting Information Fig. 5). No significant impact was observed on the M38 responses. This suggests that while M45-specific CD8**^+^** T cells are less frequent than their M38 counterparts, the actual frequency of cells is rather stable, bouncing back quickly after depletion. Whether this is also maintained through exposure to antigen through low level transcription or replication, or through other homeostatic mechanisms is not fully defined.

Taken together these data show that MHC-peptide tetramers can deliver cytotoxic molecules to virus-specific CD8^+^ T cells in vivo, deleting epitope-specific cells. Although the depletion and subsequent expansion of CD8^+^ T cells was quite specific, some increased expression of Ki67 was seen in the M45 control subset (20–30% compared to 100% in M38) without further expansion of this pool. It is possible that “off-target” effects (including binding to nontetramer specific T cells, B cells or macrophages) or toxicity could contribute to impacts on other CD8^+^ T-cell specificities, although overall these appeared to be modest compared to the impact on the target population, and no equivalent changes in memory inflation were observed using the M45 tetramer for depletion. When used in the context of persistent infections they reveal the dynamic flexibility of the memory compartment, even the very large “inflationary” responses. While transient depletion of a single epitope-specific population may be followed by rebound and/or overshoot, a prolonged depletion of M38-specific CD8^+^ T cells may be required to assess control of viral reactivation (we did not observe any viral rebound at 48 h; data not shown). However since there are other inflationary epitopes it is likely that even if prolonged depletion were possible, activity of other responses would likely contribute to control of reactivation.

It is interesting that the phenotype of inflationary populations did not change after depletion. This is surprising, since staining for Ki67 revealed the cell population is dividing rapidly in the rebound phase, but supports the idea that they are a stable, phenotypically robust population. These data are important as they reveal both the proliferative potential of the pool as well as the fact that the rebound seen is due to cell division rather than redistribution. That such cell populations are sustained at such a high level rather than recontracting is possibly explained by a relatively slow turnover of the pool, which may be maintained by regular antigen re-encounter and/or via cytokines. It is important to note, that the depletion was not complete and this raises the possibility that specific persistent clones may subsequently expand differentially; such a phenomenon may lead to redistribution of the restricted TCR usage [3] in the enlarged memory pool. A similar “overshoot” phenomenon with expansion and maintenance of the effector-memory phenotype was observed in human CMV responses following bone marrow population in a paediatric setting [14]. Given their combination of a stable, maintained phenotype and function with clear capacity to proliferate in vivo the “inflationary” CD8^+^ T-cell memory pool should be of therapeutic interest in relation to vaccine design using persistent vectors.

## Materials and methods

### Ethics statement

Mouse experiments in Oxford were performed according to UK Home Office regulations (project licence number PPL 30/2235 and 30/2744) and after review and approval by the local ethical review board at the University of Oxford.

### Mice

C57BL/6 mice were obtained from Harlan UK. Experiments were performed using age and sex matched mice.

### Viruses

MCMV strain (Strain Smith, ATCC: VR194) was used and kindly provided by Professor U. H. Koszinoswki, Department of Virology, Max von Pettenkofer Institute, Munich Germany. MCMV was propagated and titrated on NIH 3T3 cells (ECACC, UK), stored at −80°C and injected intravenously at a dose of 1 × 10^6^ pfu.

### Peptides

Peptides derived from MCMV, M38_316_–_323_ (SSPPMFRV) and M45_985_–_993_ (HGIRNASFI) were purchased from Proimmune, Oxford, UK.

### Antibodies

Antibodies were obtained from ebioscience (San Diego, USA), BD Bioscience (Oxford, UK), Biolegend (San Diego, USA), and R&D (Abingdon, United Kingdom).

### Peptide stimulation and intracellular staining

For peptide stimulation, 1 × 10^6^ cells were stimulated for 2 h at 37°C with either 10^−4^ M M38 or 10^−4^ M M45 peptide. As a positive control cells were stimulated with phorbol myristate acetate (PMA) (50 ng/mL) and ionomycin (500 ng/mL) or left untreated as a negative control. After 2 h GolgiPlug (1 μL/1 mL final concentration) BD Bioscience (Oxford, UK) was added to each well and incubated for a further 4 h at 37°C.

Intracellular staining was carried out by, fixing and permeabilized using the FOXP3 Fixation/permeabilization Kit (ebioscience). Cells were resuspended in permeablization buffer containing the appropriate amount of antibody and incubated for 30 min at 4°C.

### Isolation of BM, liver, and lung lymphocytes

Bone marrow was isolated by washing the femur shaft with PBS. Cells were passed through a 70 μm nylon filter (BD) and red cell lysis was performed. Perfused livers were passed through a 70 μm nylon filter (BD) and lymphocytes were purified by a Percoll (GE healthcare) gradient centrifugation. Lungs were minced with razor blades and incubated in PBS containing 60 U/mL DNase (AppliChem) and 170 U/mL collagenase II (Gibco) at 37°C for 45 min. Cell aggregates were dispersed by passing the digest through a 70 μm nylon filter (BD).

### Construction of tetrameric MHC class I peptide complexes

MHC class I monomers complexed with M38 (H-2Kb) and M45 (H-2Db) and produced as previously described then tetramerized by addition of streptavidin–PE (BD Bioscience, Oxford, UK), streptavidin-APC (Invitrogen, Paisley, UK) or streptavidin-saporin (Advanced Targeting Systems). Aliquots of 1 × 10^6^ cells or 100 μL of whole blood were stained using 50 μL of a solution containing tetrameric class I-peptide complexes. Staining was performed at 37°C for 20 min followed by staining with mAbs.

### Flow cytometry

Single cell suspensions were generated from the indicated organs and 1 × 10^6^ cells were incubated with the indicated mAb at 4°C for 20 min, erythrocytes were lysed with RBC Lysing Solution (BD PharMingen). Cells were analyzed using a LSRII (BD) flow cytometer and FlowJo software, gated on viable leukocytes using the live/dead fixable near-IR cell stain kit from Invitrogen (Paisley, UK).

### Statistical analyses

Statistical significance was assessed by the Mann–Whitney test using GraphPad Prism software. *p*-values < 0.05 were considered significant.

## Abbreviations

MCMV: murine cytomegalovirus

## Supporting Information

As a service to our authors and readers, this journal provides supporting information supplied by the authors. Such materials are peer reviewed and may be re-organized for online delivery, but are not copy-edited or typeset. Technical support issues arising from supporting information (other than missing files) should be addressed to the authors.

Peer review correspondence

Figure S1. Representation of the killing of specific CD8^+^ T-cells by tetramer conjugated saporin, a ribonuclease inactivating protein. Upon binding of the MHC class I tetramer to the TCR on a CD8^+^ T-cell, the tetramer will be internalized. In the lysosomal compartment it will dissociate and the saporin will travel to the ribosomes, leading to inactivation and death of the specific CD8^+^ Tcell. Figure S2. Depletion of M38-specific CD8^+^ T-cells in organs. C57BL/6 mice were infected (i.v) with 1 × 10^6^ pfu MCMV; 50 days post infection, mice were injected with either M38-teramer-saporin or M38-teramer-PE, lymphocytes harvested from the liver, lung and spleen. (A) Time course for M38-(red) and M45- (blue) specific CD8^+^ T cells after injection with M38-teramer-saporin, and M38- (orange) and M45- (light blue) specific CD8^+^ T cells after injection with M38-teramer-PE. Lymphocytes were harvested from the liver, lung and spleen at 0, 2, and 6 days post injection, stained with tetramers, and analysed by flow cytometry. (B) Representative flow cytometry plots of M38-specific CD8^+^ T cells and M45-specific CD8^+^ T cells 50 days after MCMV infection and 2 and 6 days after either M38-tetramer- saporin or M38-tetramer-PE injection. (A and B) Data represent mean percentages of live tetramer^+^ CD8^+^ T lymphocytes ((mean ± SEM) of n = 6/group and are pooled from 2 independent experiments). Figure S3. Total cell count of M38-Specific CD8^+^ T-cells after M38-tetramer-saporin injection. C57BL/6 mice were infected (i.v) with 1 × 10^6^ pfu MCMV. 50 days post infection mice were injected with M38-teramer-saporin. lymphocytes harvested and from the spleen. Data represent mean percentages of live lymphocytes ((mean ± SEM) of n = 3/group are within a single experiment and do not provide information regarding experimental reproducibility. Figure S4. Phenotype of specific CD8^+^ T-cells after M38-tetramer-saporin injection. C57BL/6 mice were infected (i.v) with 1 × 10^6^ pfu MCMV. 50 days post infection mice were injected with M38-teramer-saporin, indicated by yellow arrow. lymphocytes harvested from the spleen, liver, lung and blood, and stained with M38- and M45-Tetramer and the indicated marker. Data represent mean percentages of live tetramer^+^ CD8^+^ T lymphocytes and the indicated marker ((mean ± SEM) of n = 6/group and are pooled from 2 independent experiments). Figure S5. Depletion of M45-specific CD8^+^ T-cells. C57BL/6 mice were infected (i.v) with 1 × 10^6^ pfu MCMV; 50 days post infection, mice were injected with M45-teramer-saporin, indicated by yellow arrow; mice were bled over a time course. (A) Time course for M38- (red) and M45- (blue) specific CD8^+^ T-cells after injection with M45-teramer-Saporin, Mice were bled post injection, stained with tetramers, and analysed by flow cytometry. Mean percentages of live tetramer^+^ CD8^+^ lymphocytes are indicated. (B) Representative flow cytometry plots of M38- and M45-specific CD8^+^ T-cells, 52 and 71 days after MCMV infection (2 and 11 days after M45-tetramer-saporin injection, respectively). (A and B) Data represent mean percentages of live tetramer^+^ CD8^+^ T lymphocytes ((mean ± SEM) of n = 6/group and are pooled from 2 independent experiments).
