# Supplementary material for: Increasing inflationary T-cell responses following transient depletion of MCMV-specific memory T cells
Source: Eur J Immunol. 2014 Oct 20;45(1):113–8. doi: 10.1002/eji.201445016 (PMC4477908; doi:10.1002/eji.201445016)
Supplement: Supplementary file 2 [file eji0045-0113-sd2.pdf]

European Journal of Immunology

# Supporting Information for DOI 10.1002/eji.201445016

Stuart Sims and Paul Klenerman

## Increasing inflationary T-cell responses following transient depletion of MCMV-specific memory T cells

© 2014 WILEY-VCH Verlag GmbH & Co. KGaA, Weinheim

European Journal of Immunology

# Supporting Information
## for
DOI 10.1002/eji.201445016

Stuart Sims and Paul Klenerman

**Increasing inflationary T-cell responses following transient depletion of MCMV-specific memory T cells**

© 2014 WILEY-VCH Verlag GmbH & Co. KGaA, Weinheim

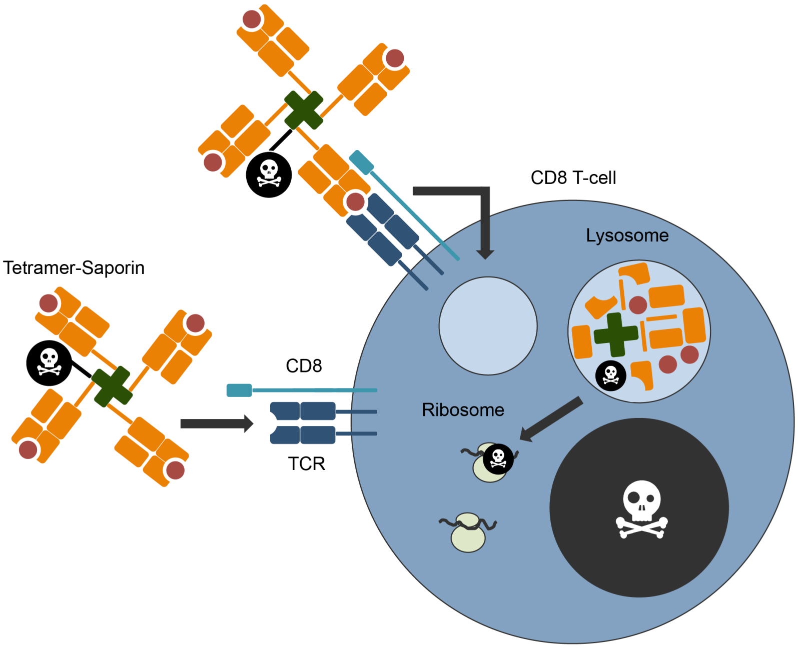


**Supporting Information Figure 1: Representation of the killing of specific CD8$^+$ T-cells by tetramer conjugated saporin, a ribonuclease inactivating protein**. Upon binding of the MHC class I tetramer to the TCR on a CD8$^+$ T-cell, the tetramer will be internalized. In the lysosomal compartment it will dissociate and the saporin will travel to the ribosomes, leading to inactivation and death of the specific CD8$^+$ T-cell.

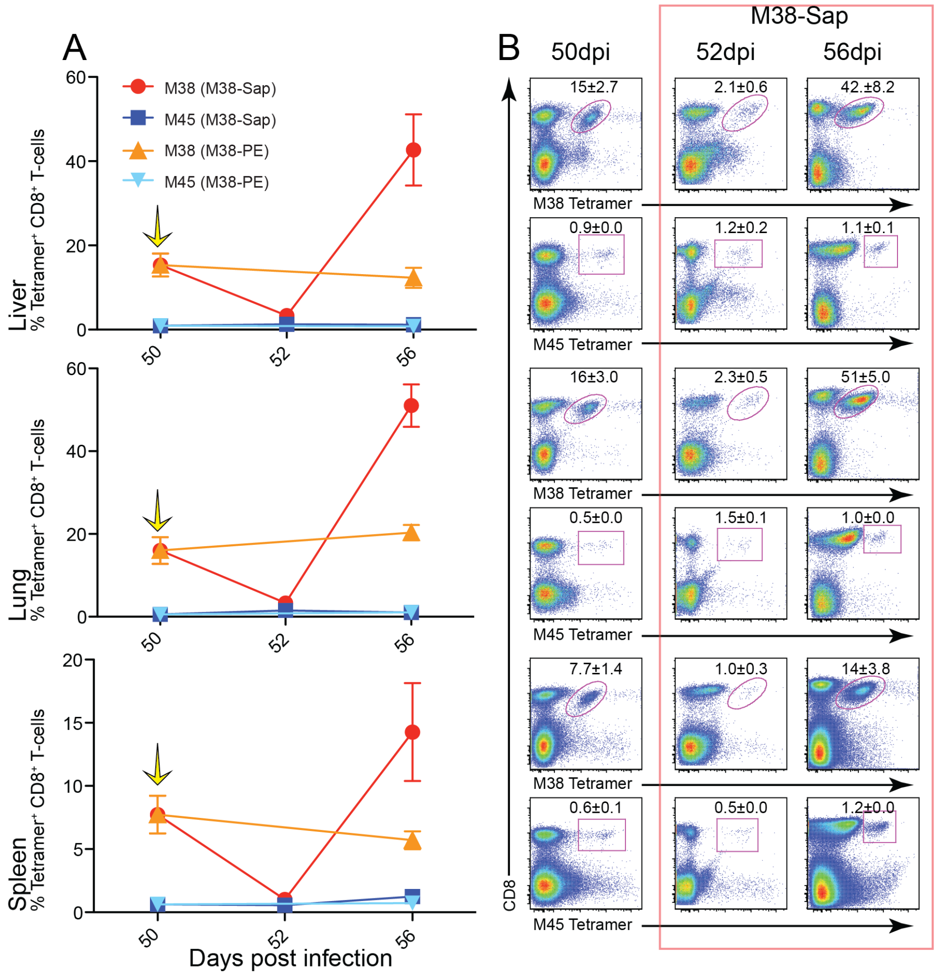


**Supporting Information Figure 2: Depletion of M38-specific CD8+ T-cells in organs**. C57BL/6 mice were infected (i.v) with 1x10$^{6}$ pfu MCMV; 50 days post infection, mice were injected with either M38-teramer-saporin or M38-teramer-PE, lymphocytes harvested from the liver, lung and spleen. (A) Time course for M38- (red) and M45- (blue) specific CD8$^{+}$ T cells after injection with M38-teramer-saporin, and M38- (orange) and M45- (light blue) specific CD8$^{+}$ T cells after injection with M38-teramer-PE. Lymphocytes were harvested from the liver, lung and spleen at 0, 2, and 6 days post injection, stained with tetramers, and analysed by flow cytometry. (B) Representative flow cytometry plots of M38-specific CD8$^{+}$ T cells and M45-specific CD8$^{+}$ T cells 50 days after MCMV infection and 2 and 6 days after either M38-tetramer- saporin or M38-tetramer-PE injection. (A and B) Data represent mean percentages of live tetramer$^{+}$ CD8$^{+}$ T lymphocytes ((mean ± SEM) of n=6/group and are pooled from 2 independent experiments).

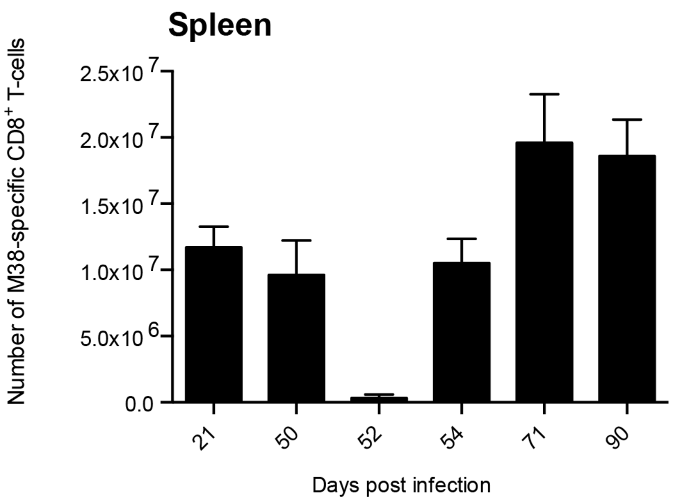


**Supporting Information Figure 3: Total cell count of M38-Specific CD8$^{+}$ T-cells after M38-tetramer-saporin injection**. C57BL/6 mice were infected (i.v) with 1x10$^{6}$ pfu MCMV. 50 days post infection mice were injected with M38-teramer-saporin. lymphocytes harvested and from the spleen. Data represent mean percentages of live lymphocytes ((mean ± SEM) of n=3/group are within a single experiment and do not provide information regarding experimental reproducibility.

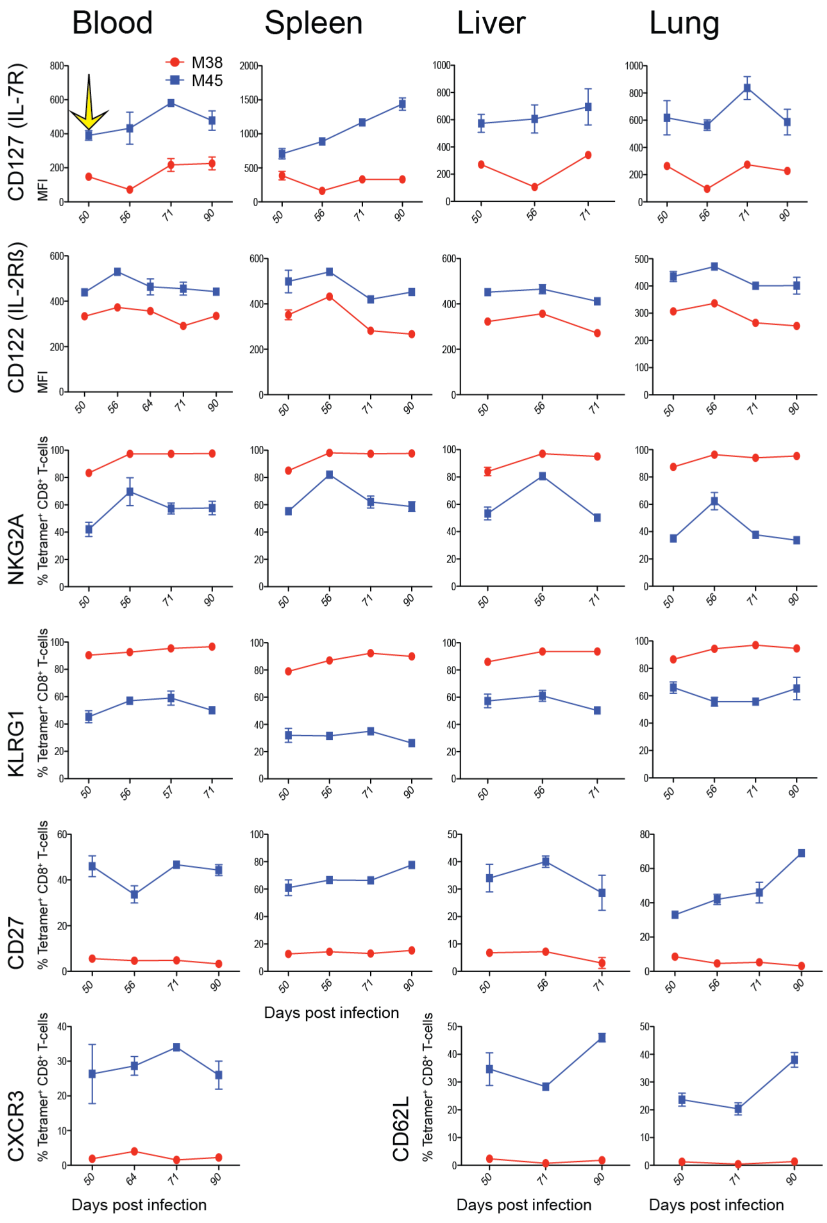


**Supporting Information Figure 4: Phenotype of specific CD8+ T-cells after M38-tetramer-saporin injection**. C57BL/6 mice were infected (i.v) with 1x$10^6$ pfu MCMV. 50 days post infection mice were injected with M38-teramer-saporin, indicated by yellow arrow**.** lymphocytes harvested from the spleen, liver, lung and blood, and stained with M38- and M45-Tetramer and the indicated marker. Data represent mean percentages of live tetramer$^+$ CD8$^+$ T lymphocytes and the indicated marker ((mean ± SEM) of n=6/group and are pooled from 2 independent experiments).

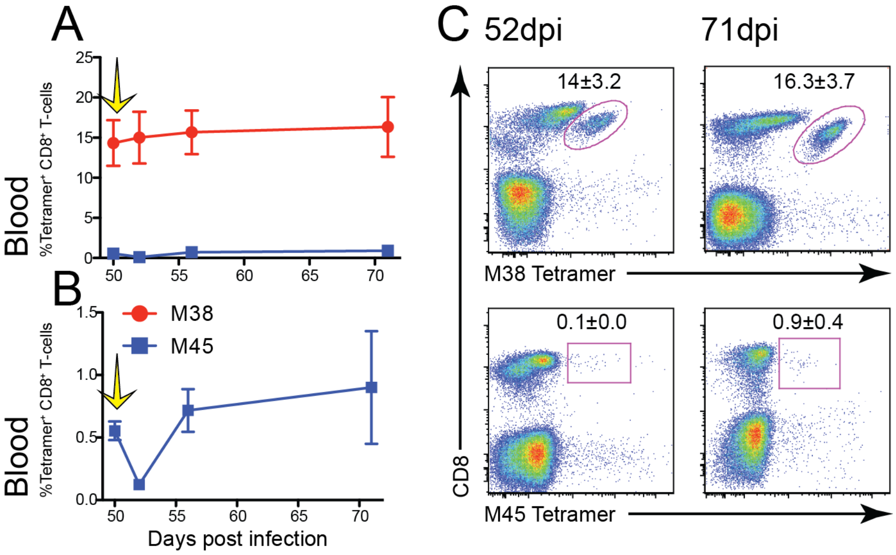


**Supporting Information Figure 5: Depletion of M45-specific CD8+ T-cells.** C57BL/6 mice were infected (i.v) with $1x10^6$ pfu MCMV; 50 days post infection, mice were injected with M45-teramer-saporin, indicated by yellow arrow; mice were bled over a time course. (A) Time course for M38- (red) and M45- (blue) specific CD8+ T-cells after injection with M45-teramer-Saporin, Mice were bled post injection, stained with tetramers, and analysed by flow cytometry. Mean percentages of live tetramer+ CD8+ lymphocytes are indicated. (B) Representative flow cytometry plots of M38- and M45-specific CD8+ T-cells, 52 and 71 days after MCMV infection (2 and 11 days after M45-tetramer-saporin injection, respectively). (A and B) Data represent mean percentages of live tetramer$^+$ CD8$^+$ T lymphocytes ((mean ± SEM) of n=6/group and are pooled from 2 independent experiments).
