# Supplementary material for: Increasing inflationary T-cell responses following transient depletion of MCMV-specific memory T cells
Source: Eur J Immunol. 2014 Oct 20;45(1):113–8. doi: 10.1002/eji.201445016 (PMC4477908; doi:10.1002/eji.201445016)
Supplement: Supplementary file 1 — Peer review correspondence [file eji0045-0113-sd1.pdf]

European Journal of Immunology

Peer review correspondence

Increasing inflationary T-cell responses following transient depletion of MCMV-specific memory T cells

Stuart Sims and Paul Klenerman

Correspondence: Peter Medawar Building for Pathogen Research, University of Oxford, Oxford OX1 3SY, UK

Review Timeline:	Submission date:	9 July 2014
	First Editorial decision:	18 August 2014
	First Revision received:	8 September 2014
	Accepted:	17 October 2014

Handling Executive Committee member: Prof. Andreas Radbruch

Please note that the correspondence below does not include the standard editorial instructions regarding preparation and submission of revised manuscripts, only the scientific revisions requested and addressed.

First Editorial Decision – 18 August 2014

Dear Dr. Sims,

Please accept my sincere apologies for the prolonged delay in processing the review of your Manuscript ID eji.201445016 entitled "Increasing CD8 T-cell memory by depleting CD8 T-cell memory", which you submitted to the European Journal of Immunology. There was a difference in opinion for which we sought additional advice.

All opinions have now been received and the comments of the referees are included at the bottom of this e-mail. Even though Ref. #1 is quite positive about your manuscript, Ref.#2 and ref.#3 have some concerns, and Ref.#2 recommended rejection. The Executive editor would like to see a revised version of your manuscript that takes into account the comments of all the referees. This revised version will be reconsidered for publication.

European Journal of Immunology

You should also pay close attention to the editorial comments included below. In particular, please edit your figure legends to follow Journal standards as outlined in the editorial comments. Failure to do this will result in delays in the re-review process.

Please note that submitting a revision of your manuscript does not guarantee eventual acceptance, and that your revision will be re-reviewed by the referees before a decision is rendered.

If the revision of the paper is expected to take more than three months, please inform the editorial office. Revisions taking longer than six months may be assessed by new referee(s) to ensure the relevance and timeliness of the data.

Once again, thank you for submitting your manuscript to European Journal of Immunology and we look forward to receiving your revision.

Yours sincerely,
Laura Soto Vazquez

On behalf of
Prof. Andreas Radbruch

Editorial Office
European Journal of Immunology
e-mail: ejied@wiley.com
www.eji-journal.eu

****

Reviewer: 1

Comments to the Author
I thought this was a very interesting paper that casts an additional layer of complexity on CMV CD8 hyperinflation in man and mouse. The only issue I have is that the resolution quality of the figures needs to be improved for publication. The technique to delete epitope specific CD8 T cells is interesting and the hyperinflation of CD8 clones post deletion is interesting. I don't agree with the conclusion that this would be good for vaccinology but that’s my opinion and does not detract from the observations. I had always assumed and still do think that the hyperinflation must be driven by MCMV somewhere but this paper finds little evidence of viral reactivation at least 2 days post depletion. In future studies it would be interesting to look where proliferating CD8s first reappeared.

Reviewer: 2

Comments to the Author

In the present study the authors depleted MCMV-specific CD8+ T cells with the help of cytotoxic MHC I-Saporin (SAP) tetramers and studied the composition of the resulting CD8+ T cell pool. M38 (H-2Kb)-SAP-tetramers were used to deplete inflationary, M38-specific CD8+ T cells. Immunodominant, M45-specific CD8+ T cells were depleted with corresponding M45 (H-2Db)-SAP-tetramers. However, the deletion of both CD8+ T cell populations was incomplete, transient and followed by a rebound effect, which was more pronounced for M38-specific than for M45-specific CD8+ T cells. The rebound phase was characterized by the transient proliferation of M38-specific CD8+ T cells. Finally, the M38-specific CD8+ T cell pool increased in size and finally became larger than before depletion. The authors suggest that this experimental approach might “reveal a novel method of inducing high CD8+ T cell responses relevant to persistent vaccine vectors”.

However, as shown in Fig. 2B, the proliferative response during the rebound phase was also detectable for M45-specific CD8+ T cells, although they should not have been affected by M38 SAP-tetramers. This might have been due to toxic side effects of SAP-tetramers (liver toxicity) as they were already reported in Ref. 9. Such side effects might further be promoted by the binding of SAP-tetramers to CD8-negative immune cells. As shown in Fig. 1A (dot plots, 50dpi, lower right quadrants) this appeared to be the case particularly at late stages of infection. These points raise doubts concerning the specificity of the treatment regimen.

Based on the strong response of M38-specific CD8+ T cells and the stability of their effector functions (Fig. 2C and D) the authors suggest that SAP-tetramers might be useful to boost anti-viral immune responses. If this would be the case, one would expect a long-term reduction of viral load in those mice treated with M38 SAP-tetramers (Fig. 2A). Unfortunately, this information is missing and it remains unclear whether the expansion of M38-specific CD8+ T cells would support viral clearance or just reflects a homeostatic response without any functional relevance.

Reviewer: 3

Comments to the Author

Major comments

Overall, this is an interesting manuscript. However, some aspects are presented in a somewhat misleading way and need to be corrected.

1.) The current title is too general and therefore misleading. The title should be chanced for example to “Increasing inflationary T cell responses upon partial MCMV-specific memory T cell depletion”.

European Journal of Immunology

2.) The focus on vaccine development – as stated for example in the abstract or in the discussion - is not really clear to me. Inflationary response are high in frequency, but it is unclear to what extend they really contribute to protection. Is a vaccine induced T cell population of 10% more protective that a population of 20%? I think such a conclusion cannot be drawn from the presented data. Without such data I would recommend to leave the “vaccine focus” completely out.
3.) The authors should not talk about “depletion of epitope-specific CD8+ T cells”, they should correctly say “partial depletion of epitope-specific CD8+ T cells” (especially in the abstract).
4.) To me the most interesting finding is that the inflationary CD8+ T cell response stays high upon partial depletion, whereas the non-inflationary response gets back to its initial frequency. How do the authors explain this? What exactly does the MHC multimer in vivo depletion do to the T cell population? Assuming that the inflationary T cell population is still polyclonal at the time of partial depletion, are certain T cells within the population more susceptible to MHC multimer depletion than others? This could be determined by changes in the TCR repertoire. Have the authors looked for that? A detailed antigen-specific TCR repertoire analysis might go beyond the scope of the study, but a basic TCR Valpha/beta staining with routinely available antibodies should be done. A clear shift in TCR Valpha/beta composition might indicate that some T cells are more susceptible to in vivo depletion (in parallel, similar experiments should be done with the non-inflationary T cell population upon partial depletion).
5.) The data looking for virus load during MCMV infection as well as shortly upon M38-specific T cell depletion are not very meaningful. The depletion is only partial and the virus load upon depletion is only followed short-term. I would suggest to completely leave out these data.

Specific comments

1.) Figure 2: Only % tetramer positive cells are shown. The authors also should calculate absolute number and show them in a figure.

<u>First Revision – authors’ response – 8 September 2014</u>

Reviewer: 1

Comments to the Author
I thought this was a very interesting paper that casts an additional layer of complexity on CMV CD8 hyperinflation in man and mouse. The only issue I have is that the resolution quality of the figures needs to be improved for publication. The technique to delete epitope specific CD8 T cells is interesting and the hyperinflation of CD8 clones post deletion is interesting. I don't agree with the conclusion that this would be good for vaccinology but thats my opinion and does not detract from the observations. I had always assumed and still do think that the hyperinflation must be driven by MCMV somewhere but this paper finds

European Journal of Immunology

little evidence of viral reactivation at least 2 days post depletion. In future studies it would be interesting to look where proliferating CD8s first reappeared.

We thank the reviewer for the comments and have submitted high resolution figures. We have in fact removed the discussion around vaccines as suggested below. The issue around the persistence and reactivation of virus is quite complex and we agree it is likely that viral antigen is involved. In general during late infection, even following depletion of single cell subsets, the levels of antigen and transcript detected are very low and it will likely depend on expression of sufficient peptide-MHC on niche cell populations in specific tissues. The suggestion of looking at the site of initial expansion is a good one and we could pursue this in future studies as there is debate in literature as to how/where memory inflation is sustained and this data could be a valuable addition.

Reviewer: 2

Comments to the Author

In the present study the authors depleted MCMV-specific CD8+ T cells with the help of cytotoxic MHC I-Saporin (SAP) tetramers and studied the composition of the resulting CD8+ T cell pool. M38 (H-2Kb)-SAP-tetramers were used to deplete inflationary, M38-specific CD8+ T cells. Immunodominant, M45-specific CD8+ T cells were depleted with corresponding M45 (H-2Db)-SAP-tetramers. However, the deletion of both CD8+ T cell populations was incomplete, transient and followed by a rebound effect, which was more pronounced for M38-specific than for M45-specific CD8+ T cells. The rebound phase was characterized by the transient proliferation of M38-specific CD8+ T cells. Finally, the M38-specific CD8+ T cell pool increased in size and finally became larger than before depletion. The authors suggest that this experimental approach might “reveal a novel method of inducing high CD8+ T cell responses relevant to persistent vaccine vectors”.

However, as shown in Fig. 2B, the proliferative response during the rebound phase was also detectable for M45-specific CD8+ T cells, although they should not have been affected by M38 SAP-tetramers. This might have been due to toxic side effects of SAP-tetramers (liver toxicity) as they were already reported in Ref. 9. Such side effects might further be promoted by the binding of SAP-tetramers to CD8-negative immune cells. As shown in Fig. 1A (dot plots, 50dpi, lower right quadrants) this appeared to be the case particularly at late stages of infection. These points raise doubts concerning the specificity of the treatment regimen.

We agree that the binding of MHC-peptide tetramers is not 100% specific – all tetramer reagents have a certain level of non-specific binding, as do antibodies. On a flow cytometry plot, of course, these can be further gated out by use of exclusion stains; in vivo there will doubtless also be binding to such FACS-

excluded cells such as macrophages, B cells etc – this might depend on the local concentrations which we do not represent in vitro. It is possible therefore that non-specific binding and toxicity due to cell death from the M38 pools may have contributed to the rebound effect seen and off-target impacts. However, the impact on M45-specific cells, which are an excellent control, being primed at the same time and co-existing in the same animal, was very modest, with only a small degree of Ki67 seen and no significant depletion or expansion of the pool, unlike the dramatic effects seen in the target M38 population. Furthermore – the reverse experiment (using an M45 tetramer) did not reveal a substantial change in the M38 pool (Figure S4).

To address the reviewer's point, we have clarified this with several changes in the text. Overall our conclusion would be (and this is consistent with the previous use of such reagents) that the depletion effect is quite specific within the CD8+ T cell pool, but we have added a comment to elaborate on the alternative impacts of the treatment via non-specific binding and toxicity.

Based on the strong response of M38-specific CD8+ T cells and the stability of their effector functions (Fig. 2C and D) the authors suggest that SAP-tetramers might be useful to boost anti-viral immune responses. If this would be the case, one would expect a long-term reduction of viral load in those mice treated with M38 SAP-tetramers (Fig. 2A). Unfortunately, this information is missing and it remains unclear whether the expansion of M38-specific CD8+ T cells would support viral clearance or just reflects a homeostatic response without any functional relevance.

As suggested below we have removed the discussion on the viral load. It is hard to know the overall impact one would expect with an increase in memory pools – if they respond to a very minor increase in transcripts both load and memory might be expanded slightly, or alternatively they might reach some new equilibrium at a later date, depending on the overall impact of the cells. The main difficulty is accurate measurement of the relevant portion of the viral antigen pool, or viral transcripts, which are according to the literature being stochastically generated at tissue sites at very low levels.

We think that regardless of the impact on the viral load long term, the finding is of relevance as the homeostasis of this pool of memory T cells has not been fully defined and differs from other populations such as resting classical memory or exhausted cells. The finding that it can be dramatically and rapidly regulated is novel and suggests more malleability in this population than previously considered. This might or might not be relevant to CMV itself but CMV-based vectors are being used (eg against SIV) and also memory inflation has been seen in response to adenoviral vectors, so it may be that boosting such populations could have functional relevance in vivo. Anyway, we have deleted the sections on vaccinology as they are not so immediately relevant to the findings here, as suggested below, and additionally removed the section on viral load, also as suggested.

European Journal of Immunology

Reviewer: 3

Comments to the Author

Major comments

Overall, this is an interesting manuscript. However, some aspects are presented in a somewhat misleading way and need to be corrected.

1.) The current title is too general and therefore misleading. The title should be chanced for example to “Increasing inflationary T cell responses upon partial MCMV-specific memory T cell depletion”.

We have modified this. The new title is “Increasing inflationary T cell responses following transient depletion of MCMV-specific memory T cells” which we hope reflects the dynamic nature of the intervention

2.) The focus on vaccine development – as stated for example in the abstract or in the discussion - is not really clear to me. Inflationary response are high in frequency, but it is unclear to what extend they really contribute to protection. Is a vaccine induced T cell population of 10% more protective that a population of 20%? I think such a conclusion cannot be drawn from the presented data. Without such data I would recommend to leave the “vaccine focus” completely out.

We have removed this throughout.

3.) The authors should not talk about “depletion of epitope-specific CD8+ T cells”, they should correctly say “partial depletion of epitope-specific CD8+ T cells” (especially in the abstract).

We have modified this through the manuscript.

4.) To me the most interesting finding is that the inflationary CD8+ T cell response stays high upon partial depletion, whereas the non-inflationary response gets back to its initial frequency. How do the authors explain this? What exactly does the MHC multimer in vivo depletion do to the T cell population? Assuming that the inflationary T cell population is still polyclonal at the time of partial depletion, are certain T cells within the population more susceptible to MHC multimer depletion than others? This could be determined by changes in the TCR repertoire. Have the authors looked for that? A detailed antigen-specific TCR repertoire analysis might go beyond the scope of the study, but a basic TCR Valpha/beta staining with routinely available antibodies should be done. A clear shift in TCR Valpha/beta composition might indicate that some T cells are more susceptible to in vivo depletion (in parallel, similar experiments should be done with the non-inflationary T cell population upon partial depletion).

The suggestion is very interesting and we have thought hard about what to do. We have not addressed this in the current manuscript as to establish such a protocol and reproduce it for new sets of memory

European Journal of Immunology

mice would take several months (>3m and with repeats likely >6m). I think given the timescale and effort involved probably it would be a better and more definitive experiment performed using TCR sequencing, as follows.

1. Previous work in the BALB/c model has shown a dominance of specific Vbeta chains, even from early stages of infection
2. In early sanger sequencing experiments following a PCR-cloning protocol on tetramer sorted cells, quite some diversity of D and J regions associated with the dominant Vb 8.1 chain (unpublished data): thus looking deeper into the clonality will be critical.
3. We have also performed a preliminary experiment using a limited set of Vb antibodies in the B6 mice using a simplified model of memory inflation (Bolinger et al JI 2013). This confirmed a dominant usage of Vb8.1, but also quite marked mouse-to-mouse variability. Given the nature of the stain, we cannot do sequential sampling from the blood of single mice, but need to take splenocytes. Thus we will need to account for this mouse-to-mouse variability in experimental design.

We are therefore establishing a deep sequencing protocol for Va and Vb based on analyses of tetramer-sorted cells with collaborators, but this is still undergoing optimization. We will I think also need to perform some rigorous controls to understand the turnover of the clones in a steady state, and how much of any change we do observe is stochastic vs how much is reproducible between mice. We have added a note in the discussion (lines 224-229) regarding this specific point. Certainly in these future experiments, the ability to partially “interrupt” memory in this way will provide an important tool to try and understand the clonal architecture and how it is maintained.

5.) The data looking for virus load during MCMV infection as well as shortly upon M38-specific T cell depletion are not very meaningful. The depletion is only partial and the virus load upon depletion is only followed short-term. I would suggest to completely leave out these data.

We have followed this advice.

Specific comments
1.) Figure 2: Only % tetramer positive cells are shown. The authors also should calculate absolute number and show them in a figure.

We have addressed this point and added a new figure S3.

Peer review correspondence

European Journal of Immunology

Second Editorial Decision – 6 October 2014

Dear Dr. Sims,

It is a pleasure to provisionally accept your manuscript entitled "Increasing inflationary T cell responses following transient depletion of MCMV-specific memory T cells" for publication in the European Journal of Immunology.

For final acceptance, please follow the instructions below and return the requested items as soon as possible as we cannot process your manuscript further until all items listed below are dealt with.

Please note that EJI articles are now published online a few days after final acceptance (see Accepted Articles: http://onlinelibrary.wiley.com/journal/10.1002/(ISSN)1521-4141/accepted). The files used for the Accepted Articles are the final files and information supplied by you in Manuscript Central. You should therefore check that all the information (including author names) is correct as changes will NOT be permitted until the proofs stage.

We look forward to hearing from you and thank you for submitting your manuscript to the European Journal of Immunology.

Yours sincerely,
Laura Soto Vazquez

on behalf of
Prof. Andreas Radbruch

Editorial Office
European Journal of Immunology
e-mail: ejied@wiley.com
www.eji-journal.eu
